# Nine-months-old infants do not need to know what the agent prefers in order to reason about its goals: on the role of preference and persistence in infants' goal-attribution

**DOI:** 10.1111/j.1467-7687.2012.01151.x

**Published:** 2012-09

**Authors:** Mikolaj Hernik, Victoria Southgate

**Affiliations:** 1 Research Department of Educational, Clinical and Health Psychology, University College LondonUK; 2 Developmental Neuroscience Unit, Anna Freud CenterLondon, UK; 3 Centre for Brain and Cognitive Development, Birkbeck, University of LondonUK

## Abstract

Human infants readily interpret others’ actions as goal-directed and their understanding of previous goals shapes their expectations about an agent’s future goal-directed behavior in a changed situation. According to a recent proposal (Luo & Baillargeon, 2005), infants’ goal-attributions are not sufficient to support such expectations if the situational change involves broadening the set of choice-options available to the agent, and the agent’s preferences among this broadened set are not known. The present study falsifies this claim by showing that 9-month-olds expect the agent to continue acting towards the previous goal even if additional choice-options become available for which there is no preference-related evidence. We conclude that infants do not need to know about the agent’s preferences in order to form expectations about its goal-directed actions. Implications for the role of action persistency and action selectivity are discussed.

## Introduction

Interpreting others’ actions as goal-directed events is one of the most extensively studied infant social-cognitive abilities. It plays a vital and indispensable role in social learning (Baldwin & Baird, 2001; Csibra & Gergely, 2007), it is evident in infants from at least the end of their first half-year of life (Csibra, 2008; Woodward, 1998), and is an ability most likely shared with other species (Rochat, Serra, Fadiga & Gallese, 2008; Wood, Glynn, Phillips & Hauser, 2007).

Much of what is currently known about how human infants interpret observed action as goal-directed comes from experimental research in which infants repeatedly witness a goal-directed action, and later their reactions to similar actions, performed under modified environmental circumstances, are assessed (Gergely, Nádasdy, Csibra & Bíró, 1995; Woodward, 1998). In a particularly influential paradigm (Woodward, 1998), infants are first shown an agent repeatedly acting on, or towards, one of two available objects. Next, after the location of the two objects is reversed, infants look longer if the agent now acts on the previously un-chosen object. According to the standard interpretation, results such as these (henceforth called the ‘Woodward-effect’) are evidence for infants’ encoding of the goals of various actions, including grasping (Woodward, 1998), pointing and poking (Bíró & Leslie, 2007; Woodward & Guajardo, 2002), gazing (Johnson, Ok & Luo, 2007), approaching (Luo & Baillargeon, 2005; Schlottmann & Ray, 2010; Shimizu & Johnson, 2004), lifting (Bíró & Leslie, 2007) or moving something (Király, Jovanovic, Prinz, Aschersleben & Gergely, 2003). Underlying this paradigm is the assumption that infants’ encoding of the goal of actions repeatedly witnessed during habituation or familiarization can guide representations of goals and actions in a different context.

Using the same paradigm, a number of recent studies have shown that infants *do not* show the Woodward-effect if the goal-object was the only potential object available to the agent during familiarization, and if there is now another potential goal-object present. The lack of a Woodward-effect in single-target versions of the paradigm was first reported by Luo and Baillargeon (2005) in 5.5-month-olds familiarized with a self-propelled box approaching a solitary object. Subsequently, similar patterns of results have been reported for infants from 3.5 to 12.5 months, the stimuli involving human as well as non-human agents, and the target-object acted upon being initially either the only one present in the scene, or the only one perceptually accessible to the agent (Luo, 2011; Luo & Baillargeon, 2007; Luo & Johnson, 2009; Bíró, Verschoor & Coenen, 2011).

These results from one-object versions of the paradigm are puzzling. After all, single-target actions are interpreted by infants as goal-directed in other paradigms (e.g. Gergely *et al*., 1995; Csibra, 2008; Southgate & Csibra, 2009). Furthermore, actions directed at a single target, like simple pursuit or approach, seem like paradigmatic examples of goal-directedness, and influence adults’ interpretations of even the most basic stimuli (Opfer 2002; Tremoulet & Feldman, 2006). Last but not least, the lack of a Woodward-effect in one-object versions of the paradigm seems incompatible with the intuition that persistent acting on an object conveys sufficient information to conclude that the object is the goal of the action (Luo & Baillargeon, 2005; Luo & Beck, 2010). Indeed, persistence was thought to be an important cue to goal-directed action (Premack & Premack, 1994; Montgomery & Montgomery, 1999) long before the seminal Woodward studies.^1^ If repeated or persistent action on an object is the basis for infant’s goal-attribution, why should the presence of a second object matter?

To date, the commonly accepted answer to this question is that a goal-attribution made on the basis of an agent choosing a particular object cannot inform what that agent might do if it subsequently has different options (i.e. object-B in addition to object-A) available (Luo & Baillargeon 2005, 2007; Carey, 2009). Had the agent had the option of object-B when it chose object-A, it may not have chosen object-A at all, and so when subsequently presented with A and B, the agent’s previous goal of pursuing object-A may change. This explanation is focused on the extent to which a goal attributed during familiarization will support infants’ expectation during test trials, when the circumstances of the action have changed. Importantly, the exact details of this change of circumstances are different in different versions of the paradigm because in the one-target version there is a change in choice-options from familiarization to test (two objects instead of one) whereas in the original two-object version there is not. Crucially, on this account it is assumed that infants did attribute a goal to the agent during familiarization, based on repeated action on the same object,^2^ but that this goal-attribution is insufficient for infants to predict what the agent should do when another object becomes available.

The implication of this view is that choice, or *preference* considerations, can constrain infant’s goal-attribution-based reasoning (Luo & Baillargeon, 2007; Luo & Beck, 2010). If infants lack evidence of agents’ preferences among available goal-objects, they would have no grounds for predicting their next action.^3^ However, this hypothesis generates a rather puzzling picture which seems implausible as a general notion of the relationship between goal- and preference-attributions. First, given that real-life environments present ever-changing sets of choice-options for which the infant would frequently lack up-to-date preference-attributions, how could infants ever benefit from their goal-attributions, and use these for the purpose of action categorization, action anticipation and social learning (Csibra & Gergely, 2007)? Furthermore, some implausible predictions follow from this proposal: understanding that A has the goal of chasing B should be insufficient for infants to form the (likely correct) prediction that A will continue to chase B even when they encounter a new agent (C), as it still remains to be discovered whether A prefers B to C. In fact, recent research has demonstrated that if infants are familiarized to an agent reaching repeatedly for the same object (A) in the context of other changing objects, they nevertheless expect the agent to continue reaching for object-A, even when it is presented in the context of a new object towards which they have no information about the agent’s disposition (Feiman, Cushman & Carey, 2011).

According to the account outlined so far, the cause of infants’ failures in one-object versions of the Woodward paradigm lies in how the circumstances of the action *change* between familiarization, when the infant attributes the goal, and test, when she generates inferences on the basis of this attributed goal. However, there is another potentially important difference between the one- and two-object versions of the paradigm, which has not been considered. Specifically, the circumstances in which the action takes place are already different during familiarization, when infants are assumed to attribute a goal. In one-object versions of the Woodward paradigm, infants observe an agent persistently acting on the only object available, whereas in the classic, two-object versions, persistent action on one object occurs in the context of another object that is not acted upon. Thus, in two-object versions, the observed action can be construed both as persistent *and* selective, whereas in the one-object version, the action can be construed only as persistent. Importantly, we do not know which of these factors – persistence, or selectivity, of action – drives goal-attribution in the original, two-object Woodward paradigm, since both are present.^4^ If selectivity of action is an important cue for goal-attribution, then its absence in the one-object version could prevent infants from attributing a goal altogether. And, if infants failed to encode the action as goal-directed then this could account for why they do not demonstrate any expectation of continued action on that object during test trials. One indication that repeated action on the same object, in the absence of other cues to goal-directedness, is *insufficient* for goal-attribution may come from another well-known paradigm for assessing infants’ attribution of goals. In the so-called efficiency paradigm (Gergely *et al*., 1995), infants observe an agent (either human or non-human) repeatedly, and efficiently, pursuing a single target. However, despite repeated action on the same object, if the agent’s action was not efficiently related to the outcome (e.g. it made a movement that was unnecessarily indirect with respect to the target), then infants did not form an expectation about the agent’s subsequent actions towards this outcome (e.g. Gergely *et al*., 1995; Csibra, 2008; Kamewari, Kato, Kanda, Ishiguro & Hiraki, 2005; Southgate & Csibra, 2009).

Summing up, there are two potentially important ways in which one- and two-object versions of the Woodward paradigm differ (different contexts of action in familiarization vs. different changes in action contexts between familiarization and test) and consequently two distinct possible reasons for the lack of a Woodward-effect in one-object versions of the task (lack of action selectivity during familiarization vs. lack of up-to-date preference information at test). These two differences are inevitably confounded in the studies that compare one- and two-object versions of the task (Luo & Baillargeon, 2005, 2007; Luo & Johnson, 2009). The aim of the current study was to elucidate which of these two differences is responsible for the lack of a Woodward-effect on one-object versions. To this end, we reasoned that, if the lack of selective action during familiarization did indeed preclude infants from attributing a goal (even when there is repeated action on the same object), then providing infants with an alternative cue to goal-attribution during familiarization should reinstate the Woodward-effect, even in the absence of information about the agent’s disposition towards the newly introduced object at test. On the other hand, if infants did attribute a goal during familiarization based on repeated action on the same object, but failed to evidence the Woodward-effect due to a lack of up-to-date preference information at test (Luo & Baillargeon, 2007), no additional cues to goal-directedness present during familiarization should lead to a Woodward-effect because infants would still lack up-to-date preference information. As previously mentioned, one cue to goal-directedness that infants are known to be able to exploit is whether an action is efficiently related to an outcome, where efficiency is judged with respect to situational constraints (Gergely & Csibra, 2003). Crucially, a direct path to an object, such as that depicted in the Woodward paradigm, typically does not include information about any situational constraints that would necessitate the action pathway taken, and so may not permit infants to make a goal-attribution based on efficiency of action. Thus, in the current study, we presented 9-month-old infants with a one-object Woodward task where, in addition to observing repeated action on the same object, we manipulated whether the observed action was efficient with respect to the outcome.

## Experiment 1

Infants were assigned to either an Experimental group or to a Control group. Infants in the Experimental group saw an agent repeatedly approach a single goal-object in an efficient manner (detouring around an obstacle which blocked direct access). We predicted that the presence of efficient action would lead infants to encode the target-object as involved in the goal of the agent’s action, and that this goal-attribution would transcend the addition of another potential goal-object at test, leading infants in this group to exhibit the typical Woodward-effect, i.e. look longer on test trials in which the agent changes its goal (New Goal trials) than on those in which it continues to approach the previously chosen object in its new location (New Path trials). Infants in the No-wall Control group saw an event in which the agent took the same pathway as in the Experimental group but this time it was not necessitated by the presence of any obstacle in the scene, such that the action could not be construed as efficiently related to the outcome (e.g. Gergely *et al*., 1995; Csibra, 2008; Kamewari *et al*., 2005; Phillips & Wellman, 2005; Southgate & Csibra, 2009). This condition mirrors the one-object version of Luo and Baillargeon (2005) as it depicts repeated action on the same object, but no additional cues to goal-directedness. Thus we expected that infants in the No-wall Control group would not exhibit a Woodward-effect at test.

### Method

#### Participants

Thirty-two 9-month-old infants (16 male; mean age: 9.1 months; range: 8.3–9.8 months) participated in Experiment 1. An additional 19 infants were excluded because of not finishing the procedure (4), fussiness (7), parental interference (2), looking times to test movies shorter than 2 s (2), experimenter error (3) and a difference in looking at the two test movies that was more than 2.5 *SD*s away from the group mean (1). Sixteen infants were assigned to each of the two conditions: Experimental (mean age = 9.1 months), and No-wall control (mean age = 9.1 months).

#### Materials

**Figure 1:**
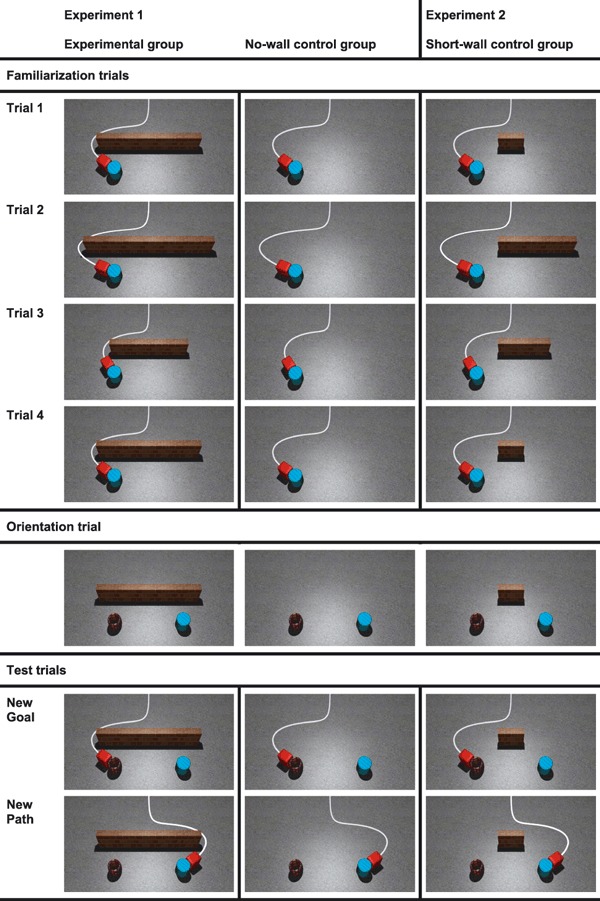
Examples of stimuli. The snapshots show static displays during which infants’ looking times were recorded. White lines indicate approach-paths of the agent and were not visible to the participants.

The stimuli were colorful 3D animations generated with *Blender* software (Figure 1). They depicted a red block (henceforth the agent) entering the scene centrally from the top of the display and approaching and stopping next to a target-object located in the foreground. For the experimental group, there was always a horizontal brick-wall standing in the middle of the screen and the agent detoured around the wall on its way to the target. The wall had three sizes across familiarization trials (in fixed order: medium, wide, narrow, medium) and the agent’s detour path varied accordingly.^5^ In each case, the duration of movement was always 4 s. On each familiarization trial, the same solitary target (blue-striped cylinder or dotted tube) stood at the same location (left or right side of the screen), and the agent took the same pathway, detouring around the side of the barrier closest to which the target-object sat. In test trials, the familiar target now stood on the opposite side of the screen, its former location now occupied by a new target-object. The wall during test was medium-sized and the agent approached either the new target (New Goal trial) or the familiar target (New Path trial). The scene was symmetrical so the agent’s left-side path was a mirror-image of the right-side path. Stimuli for the No-wall Control group were identical, but no wall was present. A single orientation trial was administered between familiarization and test which comprised the first frame of the test trial movies, and served to familiarize infants to the presence of two objects.

#### Procedure

Infants sat on their parents’ lap in a dimmed room approximately 100 cm away from a 102 × 57.5 cm plasma screen on which the stimuli were presented. Each trial was preceded by an unrelated attention-getting animation of rotating shapes. Each familiarization and test trial started with the agent approaching a target-object (approach phase, 4 s) and stopping next to it until the end of the trial (outcome phase). The four familiarization trials ended when the child looked away from the screen for more than 2 consecutive seconds after looking at the screen for 2 cumulative seconds during the outcome phase, or after 30 s of the outcome phase. The same criterion was used to terminate the orientation trial. The two test trials ended when the infant looked away for more than 2 consecutive seconds during the outcome phase. Two infants (one from each group) who watched the outcome for less than 2 seconds on a test trial were replaced.

Test trial order (New Goal first vs. New Path first), initial target-identity (cylinder vs. tube) and initial target-location (left vs. right) were counterbalanced in each group in a 2 × 2 × 2 design. Preliminary analyses found no main effects or interactions involving these factors, so they were not included in subsequent analyses.

#### Data analysis

Looking times were coded off-line by two coders: the first author and a research assistant blind to the design and hypothesis. Looking times on test trials from one-third of the babies were coded by both coders and correlated at *r* = .98, with the mean absolute difference between the codings = 550 ms. The analysis was carried out on the coding of the first author.

### Results and discussion

**Figure 2:**
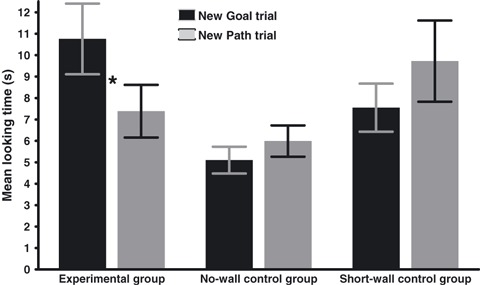
Mean looking times to New Goal and New Path test events across the three groups (Experiments 1 and 2). Error bars indicate standard errors of means. Asterisk indicates significant within-group difference.

The mean looking times during each trial for each condition are shown in Figure 2 and Table 1. As with other studies using the same paradigm (e.g. Woodward, 1998), to correct for positive skew, we applied a logarithmic transformation on the looking times before parametric analyses. Infants were very attentive during the approach-phase of the familiarization trials: only on six trials (5%) from six different participants was the agent’s approach not attended to 100% of the time. Data from the outcome phases of the familiarization trials were entered into a 4 (Trial: 1, 2, 3, 4) × 2 (Group: experimental, control) ANOVA which revealed no effect of Group [*F*(1, 29) = 2.13, *p* = .155] nor interaction [*F*(3, 87) = .438, *p* = .73]. It did, however, reveal a main effect of Trial [*F*(3, 87) = 5.14, *p* < .003] due to a significant linear trend decreasing across familiarization trials [*F*(1, 29) = 9.37, *p* < .005]. Similarly, the two groups did not differ in their looking times to the Orientation trial [*t*(30) = .46, *p* = .65]. Altogether these analyses show that both groups received equivalent exposure to familiarization and orientation events before watching test trials.

**Table 1:** Mean looking times (and standard deviations) to each trial across the three groups (Experiments 1 and 2)

	Familiarization					Test	
			
	1	2	3	4	Orientation	New Goal	New Path
Experiment 1							
Experimental	13.65 (7.88)	9.93 (7.05)	12.03 (7.41)	8.97 (8.04)	13.39 (7.59)	10.76 (6.6)	7.38 (4.91)
No-wall Control	10.70 (7.24)	7.82 (5.96)	7.35 (5.40)	6.53 (5.91)	11.70 (5.95)	5.1 (2.51)	5.99 (2.92)
Experiment 2							
Short-wall Control	11.6 (7.19)	10.39 (8.53)	10.01 (7.37)	6.66 (7.32)	10.98 (4.45)	7.55 (4.49)	9.72 (7.58)

No infant looked away from the screen during the agent’s approach on test trials. Log-transformed looking times towards the outcome of test trials were first analyzed using a 2 × 2 ANOVA with Group (experimental, control) as a between-subjects factor and Test event (New Goal, New Path) as a within-subjects factor. This analysis revealed a significant main effect of Group [*F*(1, 30) = 5.5, *p* = .026] and, crucially, a significant interaction between Group and Test event [*F*(1, 30) = 5.75, *p* = .023], but no main effect of Test event [*F*(1, 30) = .72, *p* = .4]. Planned comparisons revealed that the experimental group looked significantly longer during the New Goal than during the New Path trials [*F*(1, 30) = 5.26, *p* = .029] whereas the No-wall Control group did not [*F*(1, 30) = 1.2, *p* = .28]. Non-parametric tests further support this conclusion: in the experimental group 12 out of 16 infants looked longer during New Goal trials [*Z* = 2.12, *p* = .034, Wilcoxon two-tailed], while in the No-wall Control group, only seven out of 16 infants showed this pattern [*Z* = .98, *p* = .35, Wilcoxon two-tailed].

In order to make sure that these different looking–time patterns during test trials were not caused by some uncontrolled differences in earlier attention to the new goal-object itself, cumulative lengths of fixations (in seconds) during the Orientation trial were coded separately for the new object [Experimental group: *M* = 6.1, *SD* = 3.27; No-wall Control: *M* = 5.53, *SD* = 3.76] and for the familiar object [Experimental group: *M* = 5.04, *SD* = 3.76; No-wall Control: *M* = 4.74, *SD* = 2.63]. They were log-transformed and entered into a 2 (Group: experimental, control) × 2 (Target: familiar, new) ANOVA, which found no significant effects or interactions, thereby ruling out this low-level explanation of looking patterns in test trials.

Consistent with our prediction, infants in the Experimental group did exhibit the Woodward-effect on a one-object version of the paradigm, when provided with additional cues to goal-attribution during familiarization. Specifically, the Experimental group, but not the No-wall Control group looked significantly longer on new goal than old goal test trials, suggesting that infants in the Experimental group attributed to the agent the goal of approaching the target-object during familiarization, and expected it to continue to pursue the same goal even when presented in the context of a new potential target. Crucially, the lack of information about the agent’s preferences among the objects at test did not prevent infants from using this previously attributed goal to generate predictions in a new context. These results strongly suggest that the reason why infants failed to exhibit the Woodward-effect on previous one-object versions of the paradigm (e.g. Luo & Baillargeon, 2005, 2007) and in our No-wall Control condition is not because the presence of a new object of unknown preference status renders previous goal-attributions irrelevant, but because the lack of cues to goal-attribution during familiarization prevented infants from attributing a goal in the first place.

One objection to our explanation for the enduring goal-attributions of infants in our Experimental group is that the obstacle itself may have been interpreted by infants as a potential target, and, since the agent always approached the object (and detoured past the obstacle), perhaps this action was interpreted as selective by infants. If this was the case, and selectivity of action is a cue to goal-attribution, it may be that it is this cue, rather than the efficiency of action, that led infants to attribute a goal to the agent. Note that even if this was the case, the results would still be inconsistent with the explanation that a lack of preference information can constrain goal-attribution. Even if the infant interpreted the agent as preferring the target to the obstacle, this still does not tell the infant whether the agent would prefer the target over the new object presented alongside the target at test. Experiment 2 was designed to test whether the presence of the wall in the movies watched by the Experimental group in Experiment 1 facilitated performance because it helped infants encode actions as *efficient* and goal-directed or because it helped them encode the actions as *selective* and hence goal-directed.

## Experiment 2

If the presence of an obstacle around which the agent detours in Experiment 1 provided infants with evidence of selectivity of action and it was this, rather than evidence of efficiency of action, that led them to generate a goal-attribution, the presence of any obstacle, even if it was not accompanied by efficient action, should lead infants to generate the same expectation of continued action on the same object at test. Thus, in Experiment 2, we presented a new group of infants with events in which, although a barrier was present, the action observed by infants was not necessitated by this barrier. Specifically, in Experiment 2, although the agent detoured around the wall, the extent of the detour was unnecessary considering the width of the wall (Figure 1) and so could not be interpreted as efficient. If infants in Experiment 1 attributed a goal to the agent based on selectivity of action (i.e. acting on one object rather than another) during familiarization rather than efficiency of action, we should expect that they will do the same in Experiment 2, and subsequently expect the agent to continue pursuing the previously approached object at test. However, if it was the efficiency of the observed action in Experiment 1 that led infants to generate a goal-attribution and an expectation that the agent would continue to approach the same goal even in the presence of a new object, then the inability to efficiently relate the action to the outcome in Experiment 2 should prevent infants from attributing a goal during familiarization and consequently from forming any particular expectation about the agent’s actions at test.

### Method

#### Participants

Sixteen 9-month-old infants (8 female, mean age: 9 months, range: 8.3–9.8 months) were assigned to a new Short-wall Control group. An additional six babies were tested but excluded either because they had looking times to test movies that were shorter than 2 s (5) or because of equipment failure (1).

#### Materials and procedure

The procedure for the Short-wall Control group was identical to that of the Experimental group in Experiment 1 except for the size and position of the wall featured in the stimuli (see Figure 1). Infants in Experiment 2 saw the exact same movement pathways (width and direction) as infants in the Experimental group of Experiment 1, the only difference between these two conditions being that the wall depicted in the Short-wall Control group was always short and did not require the detour that the agent took.

#### Data analysis

Looking times on all trials were coded off-line by the first author. Looking times on test trials from half of the babies were also coded off-line by a research assistant blind to the design and hypothesis. The two codings correlated at *r* = .99, with the mean absolute difference = 170 ms. Again, the coding of the first author was used for subsequent analysis.

### Results and discussion

The mean looking times from Experiment 2 are presented in Table 1. As in Experiment 1, raw looking times were log-transformed prior to parametric analyses in order to approximate a normal distribution. No significant main effects or interactions involving test trial order, initial target-identity, or initial target-location were found.

As in Experiment 1 looking times to the familiarization events showed a significant linear decrease across familiarization [*F*(1, 15) = 5.74, *p* < .03], and this pattern did not differ from that observed in each group of Experiment 1 [highest *F*(1, 44) = .93, *p* = .33]. Similarly, the Short-wall group’s attention to the orientation trial was not different from infants in Experiment 1 [highest *t*(30) = .78, *p* = .44]. As in Experiment 1, neither of the target-objects in orientation was attended to longer than the other [*t*(15) = 1.29, *p* = .22].

As in Experiment 1, all approach phases of test trials were attended to 100% of the time. A paired-samples *t*-test revealed that, unlike the Experimental group of Experiment 1, the Short-wall group of Experiment 2 did not look longer at the New-Goal test event (in fact only eight of 16 babies showed such looking pattern). Instead, infants in the Short-wall group looked equally at both test events [t(15) = .69, *p* = .49]. Combining the results from the two experiments, a 3 (Group: Experimental, No-wall and Short-wall) × 2 (Test Event: New Goal vs. Old Goal) factor ANOVA revealed only significant simple interactions for contrasts involving the Experimental and No-wall groups [*F*(1, 45) = 4.58, *p* = .038], as well as the Experimental and Short-wall groups [*F*(1, 45) = 4.13, *p* = .048], consistent with the conclusion that the Experimental group indeed responded to test events differently from each Control group. Specifically, only the Experimental group looked longer at the New-Goal than New Path test event. No other significant effects or interactions were found.

Overall, the results of Experiment 2 suggest that the wall was not regarded by infants as a potential goal-object which could enable them to interpret the action upon the target-object as a selective action. Rather, the presence of a Woodward-effect in the Experimental group, and the lack thereof in the Short-wall group suggest that it was the efficiency of action during familiarization that enabled goal-attribution, which endured the addition of a potential new target at test.

## General discussion

The fact that infants can structure observed actions in terms of goals is incontrovertible, and indeed the ability to do so would be a prerequisite for social learning (Csibra & Gergely, 2007). What is less clear is *how* infants are able to interpret actions as goal-directed, and how these attributions could support their subsequent social reasoning. Based on findings that infants do not expect an agent to continue to pursue a previously chosen goal-object if that agent subsequently encounters other potential goals, it was proposed that goal-attributions are suspended in the absence of knowledge about an agent’s preferences among currently available goal-objects (Luo & Baillargeon, 2005, 2007). This hypothesis implies that goal-attributions could not play a fundamental role in predicting an agent’s behavior, since it is likely that there will often be potential targets appearing for which the infant might lack up-to-date preference information. For example, on this view, attributing to person A the goal of chasing person B would be suspended if these two people were subsequently observed running through a crowd of people for whom the observer had no knowledge of the agent’s disposition towards. However, the data presented here, showing that 9-month-old infants *do* expect an agent to continue to pursue a previously chosen object in the presence of new objects, if the action they had observed was efficiently related to the outcome, contradicts that view, suggesting instead that infants’ performance on those tasks reflected a failure to attribute any goal in the first place. By providing infants with a known cue to goal-attribution, we were able to show that infants exhibit the Woodward-effect despite lacking up-to-date preference or dispositional information concerning other objects (see also Bíró*et al*., 2011; Luo 2011).^6^

This finding has theoretical implications that deserve further exploration. Specifically, it suggests that the cue that infants exploit in order to attribute a goal on the standard two-object Woodward paradigm is not repeated action on an object, but rather the selectivity of the action. If repeated action was sufficient, it should result in the same expectation of continued action on the previously chosen object (even in the presence of new potential targets) that efficiency was able to generate. The assumption that a repeated reach to a solitary object is sufficient for goal-attribution has its origins in the suggestion that persistence is a good cue for goal-attribution (Premack & Premack, 1994). If an agent demonstrates continued effort to attain an outcome, a good interpretation is that this outcome was the goal of her action, and indeed young children do exploit persistence in order to interpret an action as goal-directed (Montgomery & Montgomery, 1999). This notion of persistence is assumed to be at the core of the Woodward paradigm: infants observe an agent repeatedly acting on or towards the same target-object and interpret her action as directed towards the goal of obtaining the object (e.g. Luo & Beck, 2010). Why would it not also be sufficient for goal-attribution in the one-target version (Luo & Baillargeon, 2005, 2007)? One possibility is that the actions presented to infants were not variable enough to be considered evidence of persistence. Premack and Premack argue that one caveat to the importance of persistence for interpreting an action as goal-directed is that the repeated acts must not be repeated perfectly, and indeed imperfect repetition over trials (e.g. an agent jumping at varying heights each time) has been shown to crucially affect infants’ and young children’s ability to attribute goals in other studies (e.g. Bíró & Leslie, 2007; Csibra, 2008; Montgomery & Montgomery, 1999). However, in the Woodward paradigm, infants are typically familiarized to an identical action on each trial and perhaps this lack of variation may have been a threat to goal-attribution. Further evidence that infants do not attribute a goal to a repetitive direct reach towards a single target is provided by Bíró*et al*. (2011).

While the current study provides another demonstration that efficiency of action is an important cue enabling infants to make sense of novel or ambiguous actions^7^ (Brandone & Wellman, 2009; Csibra, Bíró, Koós & Gergely, 2003; Luo, 2010; Southgate & Csibra, 2009), it also highlights a new cue that has not previously been considered to be important in goal-attribution; that of selective action. In addition to being a reliable cue towards the goal, action’s selectivity (i.e. the fact that that action is directed at one object relative to another) could enable infants to interpret that action as reflecting an agent’s preference or choice. In this sense, preference information (conveyed by selective actions) may indeed have an impact on processing of goals. But the relationship between goals and preferences implied by our study is quite different from that suggested by Luo and Baillargeon (2005, 2007). Rather than constraining or modulating inferences based on *previously* attributed goals, our data imply that preference-related information is more likely to be a factor in generating goal-attribution. Taken together with previous research (Bíró & Leslie, 2007; Király *et al*., 2003; Luo & Baillargeon, 2005), our data point to multiple cues which young infants can flexibly make use of in order to interpret an action as goal-directed. Whereas both efficiency and selective action may serve as cues to goal-attribution, it is also important to note that they are not in themselves sufficient cues for younger infants in the absence of variability of action (Bíró & Leslie, 2007; Csibra, 2008). How these cues interact, and whether some serve goal-attribution better than others, is an open question. For example, would an *inefficient* action performed selectively on a target result in goal-attribution? A recent study presented subjects with an actor who changed his mind during a reach towards one object and reached towards the other object (Carter, Hodgins & Rakison, 2011; cf. Verschoor & Bíró, 2011). While this kind of action would lack efficiency information (since the observed action detoured away from the object that was ultimately the goal), subjects may nevertheless have been able to use the selectivity of action in order to interpret the action as goal-directed.

While the Woodward paradigm is one of the most commonly used tools for investigating the psychological constructs that infants operate with, it has been taken for granted that it reflects goal-attribution based on detection of persistence in action (e.g. Luo & Beck, 2010). The current study suggests that selectivity may be the more important cue towards the goal in this paradigm. It also shows that infants do not need to know about the agent’s current preferences in order to benefit from inferences based on the previously attributed goal. Preferences expressed in selective action are more likely to generate, rather than constrain, infants’ goal-attribution.

## Footnotes

1 Premack and Premack (1994) write ‘whenever an object … persistently directs its actions towards a single item, a human observer attributes a goal to the object’.

2 Luo and Baillargeon (2007, p. 491) state: ‘The infants *no doubt* attributed to the agent the goal of contacting object-A, since the agent repeatedly approached this object across trials. However, when object-B was introduced, the infants had no basis for predicting how the agent would act: it might again approach object-A, or it might now approach object-B’ (italics added, see also e.g. Song & Baillargeon, 2007, p. 82).

3 Luo and Baillargeon (2005, p. 605) state: ‘the infants in the control [i.e. one-target] condition realized at the start of the test trials that they had no information as to whether the box would prefer the cone or the novel cylinder, and so could make no prediction about its behavior.’

4 A further, alternative, way in which infants were hypothesized to be inferring a goal in the Woodward paradigm was based on the familiarity of the action (Woodward, 1999). While familiarity does appear to modulate goal-attribution, the findings of Luo and Baillargeon (2005) confirm that it is not familiarity itself that is the crucial cue for goal-attribution on the Woodward paradigm (see also Bíró & Leslie, 2007; Király *et al*., 2003).

5 Variability of the action path during familiarization was included as previous studies have shown that infants require such additional cues to agency when events include non-human agents (e.g. Bíró & Leslie, 2007; Csibra, 2008).

6 Ad hoc modifications are of course possible which would allow one to maintain that groups failing one-target versions of Woodward’s goal-attribution task nevertheless attribute goals to one-target actions. Additional behavioral cues may somehow strengthen encoding of the goal which is attributed even in their absence (Luo, 2011). Or they may facilitate encoding of the goal in more specific terms (e.g. the goal is to approach *this* object), while in their absence the goal may be still encoded, but more broadly (e.g. the goal is to approach *an* object). We thank an anonymous reviewer for pointing out this logical possibility.

7 To our knowledge the present study is also the first to document that infants of this age require so little exposure to a completely novel action in order to evaluate its efficiency: just four 4-seconds-long approach-actions by an unfamiliar inanimate agent provided enough relevant information for the 9-month-olds in the Experimental group (Experiment 1). Moreover, this study also shows that actions watched by this group must have been encoded as efficiently related to the goal of approaching a *particular* goal-object.

## References

[b1] Baldwin DA, Baird JA (2001). Discerning intentions in dynamic human action. Trends in Cognitive Sciences.

[b2] Bíró S (2012). The nature of infants’ goal representation: commentary on Hernik and Southgate. Developmental Science.

[b3] Bíró S, Leslie AM (2007). Infants’ perception of goal-directed action: development through cue-based bootstrapping. Developmental Science.

[b4] Bíró S, Verschoor S, Coenen L (2011). Evidence for a unitary goal concept in 12-month-old infants. Developmental Science.

[b5] Brandone A, Wellman H (2009). You can’t always get what you want: infants understand failed goal-directed actions. Psychological Science.

[b6] Carey S (2009). The origin of concepts.

[b7] Carter EJ, Hodgins JK, Rakison DH (2011). Exploring the neural correlates of goal-directed action and intention understanding. NeuroImage.

[b8] Csibra G (2008). Goal-attribution to inanimate agents by 6.5-month-old infants. Cognition.

[b9] Csibra G, Gergely G (2007). ‘Obsessed with goals’: functions and mechanisms of teleological interpretation of actions in humans. Acta Psychologica.

[b10] Csibra G, Bíró S, Koós O, Gergely G (2003). One-year-old infants use teleological representations of actions productively. Cognitive Science.

[b11] Feiman R, Cushman FA, Carey SE (2011). Infants fail to represent a negative goal, but not a negative event.

[b12] Gergely G, Csibra G (2003). Teleological reasoning in infancy: the naive theory of rational action. Trends in Cognitive Sciences.

[b13] Gergely G, Nádasdy Z, Csibra G, Bíró S (1995). Taking the intentional stance at 12 months of age. Cognition.

[b14] Johnson SC, Ok S, Luo Y (2007). The attribution of attention: nine-month-olds’ interpretation of gaze as goal-directed action. Developmental Science.

[b15] Kamewari K, Kato M, Kanda T, Ishiguro H, Hiraki K (2005). Six-and-a-half-month-old children positively attribute goals to human action and to humanoid-robot motion. Cognitive Development.

[b16] Király I, Jovanovic B, Prinz W, Aschersleben G, Gergely G (2003). The early origins of goal-attribution in infancy. Consciousness and Cognition.

[b17] Kuhlmeier VA, Robson SJ (2012). Diagnosing goal attribution: commentary on Hernik and Southgate. Developmental Science.

[b18] Luo Y (2010). Do 8-month-old infants consider situational constraints when interpreting others’ gaze as goal-directed action?. Infancy.

[b19] Luo Y (2011). Three-month-old infants attribute goals to a non-human agent. Developmental Science.

[b20] Luo Y, Baillargeon R (2005). Can a self-propelled box have a goal? Psychological reasoning in 5-month-old infants. Psychological Science.

[b21] Luo Y, Baillargeon R (2007). Do 12.5-month-old infants consider what objects others can see when interpreting their actions?. Cognition.

[b22] Luo Y, Beck W (2010). Do you see what I see? Infants’ reasoning about others’ incomplete perceptions. Developmental Science.

[b23] Luo Y, Choi Y-J (2012). Infants attribute to agents goals and dispositions. Developmental Science.

[b24] Luo Y, Johnson SC (2009). Recognizing the role of perception in action at 6 months. Developmental Science.

[b25] Montgomery DE, Montgomery DA (1999). The influence of movement and outcome on young children’s attribution of intention. British Journal of Developmental Psychology.

[b26] Opfer JE (2002). Identifying living and sentient kinds from dynamic information: the case of goal-directed versus aimless autonomous movement in conceptual change. Cognition.

[b27] Phillips AT, Wellman HM (2005). Infants’ understanding of object-directed action. Cognition.

[b28] Premack D, Premack AJ, Hirschfeld LA, Gelman SA (1994). Moral belief: form versus content. Mapping the mind: Domain specificity in cognition and culture.

[b29] Rochat M, Serra E, Fadiga L, Gallese V (2008). The evolution of social cognition: goal familiarity shapes monkeys’ action understanding. Current Biology.

[b30] Schlottmann A, Ray E (2010). Goal-attribution to schematic animals: do 6-month-olds perceive biological motion as animate?. Developmental Science.

[b31] Shimizu YA, Johnson SC (2004). Infants’ attribution of a goal to a morphologically novel agent. Developmental Science.

[b32] Song H, Baillargeon R (2007). Can 9.5-month-old infants attribute to an actor a disposition to perform a particular action on objects?. Acta Psychologica.

[b33] Southgate V, Csibra G (2009). Inferring the outcome of an ongoing novel action at 13 months. Developmental Psychology.

[b34] Tremoulet PD, Feldman J (2006). The influence of spatial context and the role of intentionality in the interpretation of animacy from motion. Perception and Psychophysics.

[b35] Verschoor S, Bíró S (2011). Primacy of information about means selection over outcome selection in goal attribution by infants. Cognitive Science.

[b36] Wood JN, Glynn DD, Phillips BC, Hauser MD (2007). The perception of rational, goal-directed action in non-human primates. Science.

[b37] Woodward AL (1998). Infants selectively encode the goal object of an actor’s reach. Cognition.

[b38] Woodward AL (1999). Infants’ ability to distinguish between purposeful and non-purposeful behaviors. Infant Behavior and Development.

[b79] Woodward AL (2009). Infants’ grasp of others’ intentions. Current Directions in Psychological Science.

[b40] Woodward AL, Guajardo JJ (2002). Infants’ understanding of the point gesture as an object-directed action. Cognitive Development.

